# A Review of Physics-Informed and Data-Driven Surrogate Models for Power Transformer Fault Diagnosis

**DOI:** 10.3390/s26155007

**Published:** 2026-08-06

**Authors:** Guangfen Wan, Kai Yang, Fei Xiong, Jun Chen

**Affiliations:** 1 School of Electrical and Electronic Engineering, Huazhong University of Science and Technology, Wuhan 430074, China; wangf@cnooc.com.cn (G.W.); hust_xf@126.com (F.X.); 2 China National Offshore Oil Corporation Ltd., Beijing 100010, China; 3 College of Electrical Engineering and Control Science, Nanjing Tech University, Nanjing 211816, China; 202310007473@njtech.edu.cn

**Keywords:** power transformer, fault diagnosis, surrogate model, neural network

## Abstract

Power transformers are critical components of modern power grids, and their operational reliability directly affects power system security, stability, and continuity. With the increasing intelligence and complexity of power systems, condition monitoring and fault diagnosis of transformers have received growing attention. However, conventional diagnostic methods often face limitations such as complex modeling procedures, high computational costs, weak adaptability, and insufficient generalization under nonlinear and coupled operating conditions. In recent years, surrogate models have emerged as effective tools for transformer fault diagnosis because of their advantages in high-dimensional nonlinear mapping, rapid prediction, and data-driven approximation. This paper systematically reviews the research progress of surrogate models in transformer fault diagnosis and establishes a classification framework from the perspectives of model types, modeling strategies, data sources, and application scenarios. The principles, applicable conditions, and performance characteristics of representative surrogate models are comparatively analyzed. Furthermore, the advantages and limitations of different surrogate modeling approaches are discussed in terms of diagnostic accuracy, stability, generalization ability, interpretability, and computational efficiency. Although surrogate models show strong potential for intelligent transformer fault diagnosis, challenges remain in small-sample learning, data quality dependence, model interpretability, and cross-condition generalization. Future research should focus on multi-source information fusion, integration of physical mechanisms with data-driven learning, lightweight intelligent modeling, and standardized evaluation systems. This review aims to provide methodological guidance and technical references for the development of reliable, efficient, and interpretable transformer fault diagnosis methods.

## 1. Introduction

Power transformers are critical assets in modern power systems, where they support energy conversion, voltage regulation, grid reliability, power quality, and system stability [1]. During long-term operation, transformers are exposed to coupled electromagnetic, thermal, and mechanical stresses, together with load fluctuations and environmental disturbances [2]. These stresses can initiate insulation aging, partial discharge (PD), winding deformation, overheating, and other fault processes [3]. The increasing penetration of renewable energy sources and power-electronic devices further increases the variability and uncertainty of transformer operating conditions [4]. Fault diagnosis of transformers has consequently become a key task for reliable power-system operation. Severe transformer failures can damage expensive equipment, interrupt power supply, and trigger wider system-level risks. Accurate, efficient, and early diagnosis is therefore essential for condition-based maintenance and safe operation of modern power grids [5].

Transformer fault diagnosis has gradually evolved from periodic offline testing toward online condition monitoring and data-driven evaluation during operation. Conventional diagnostic practices mainly relied on measurements obtained during scheduled inspections or maintenance activities, such as preventive electrical tests [6,7], oil-sampling-based dissolved-gas analysis (DGA) [8,9], and vibration or acoustic analysis [10,11]. These methods remain effective for identifying specific fault signatures, but their diagnostic reliability may be affected by single-source information, fixed threshold criteria, and expert-dependent interpretation, especially when indicators fall near critical decision intervals [12,13]. Environmental interference, measurement uncertainty, and operator-related factors can further increase the risk of misclassification [14]. With the development of sensing and monitoring technologies, transformer diagnosis has increasingly shifted toward the continuous acquisition of operational signals, enabling fault assessment under online operating conditions rather than only after periodic maintenance tests. In parallel, numerical simulation methods, especially the Finite-Element Method (FEM), have been introduced to analyze electromagnetic–thermal–mechanical coupling mechanisms and fault evolution processes [15,16]. Although high-fidelity simulation can improve mechanism interpretation, its complex modeling procedure and high computational cost generally make it more suitable for offline analysis, model validation, and data generation than for direct online diagnosis or real-time prediction. Therefore, the practical applicability of a diagnostic method depends not only on the algorithmic model itself, but also on its data-acquisition mode, namely whether the input data are continuously recorded by online sensors during transformer operation or periodically collected through routine offline tests.

Surrogate models provide an efficient way to link transformer diagnostic data, physical responses, and fault-state assessment [17]. In transformer fault diagnosis, they can be understood as simplified computational models that approximate the mapping among monitoring signals, operating conditions, physical quantities, and fault states, thereby supporting rapid diagnosis, state prediction, and mechanism-assisted assessment [18]. Shadab et al. developed a Gaussian-process surrogate model for top-oil temperature prediction and thermal-parameter estimation, and further combined it with a thermal–electrical model to evaluate hotspot temperature and loss of life under model and measurement uncertainties [19]. Aizpurua et al. integrated data-driven forecasting, uncertainty modeling, and particle filtering for transformer insulation lifetime prediction, showing how machine learning-based surrogate modeling can improve remaining-life assessment using field data [20]. Tang et al. further introduced physics-informed neural networks to reconstruct and predict the temperature field of oil-immersed transformers by embedding heat-transfer equations and boundary constraints into the learning process [21]. These studies indicate that surrogate modeling in transformer fault diagnosis is moving from task-specific approximation toward a more integrated diagnostic framework that combines data representation, physical knowledge, and efficient inference. This development enhances the ability of surrogate models to support rapid fault assessment, state prediction, and mechanism-related analysis. However, their practical application is still constrained by limited and imbalanced fault samples, scenario-dependent generalization, insufficient coupling between learned features and physical mechanisms, and a lack of systematic validation across different transformer types, operating conditions, and field environments.

Recent studies indicate that surrogate models are becoming an important tool for transformer fault diagnosis by providing efficient representations between diagnostic information, physical responses, and fault states. Their application has gradually expanded from empirical fault discrimination to nonlinear state prediction, multi-source feature fusion, and mechanism-assisted assessment. This review analyzes transformer fault characteristics to identify diagnostic requirements from electrical, thermal, mechanical, and insulation-related perspectives, and further organizes surrogate modeling methods according to their representation mechanisms. By taking surrogate modeling as the main analytical thread, this review connects fault phenomena, diagnostic data, model representation, and engineering application within a unified framework. This organization clarifies how different models simplify complex transformer fault relationships and reveals their technical advantages, applicable boundaries, and remaining challenges in intelligent transformer diagnosis.

## 2. Review Methodology

This work constitutes a narrative review, rather than a systematic review or quantitative meta-analysis. The primary aim is to deliver a technically representative and critical synthesis of surrogate models and relevant modeling approaches for power transformer fault diagnosis, instead of comprehensively retrieving all the eligible literature or conducting statistical aggregation of reported findings. Literature retrieval mainly targeted peer-reviewed English journal articles published between 2016 and 2026, using the Web of Science Core Collection. Key earlier publications were also incorporated if they laid fundamental theoretical foundations for transformer fault mechanisms, diagnostic methodologies, or the development of surrogate models. After removing duplicate records and re-evaluating the relevance of each document, a total of 95 independent publications spanning 2012–2026 were selected, among which 90 papers were published within the period 2016–2026.

Search keywords were structured into three categories: research object, diagnostic task, and modeling approach. Terms associated with research objects contained power transformer, oil-immersed transformer, dry-type transformer, distribution transformer, converter transformer, transformer winding, and transformer insulation. Diagnostic keywords covered fault diagnosis, condition monitoring, partial discharge, dissolved-gas analysis, thermal faults, hotspot temperature, insulation aging, winding deformation, frequency response analysis, vibration, acoustic signals, and remaining useful life. Modeling-related keywords included surrogate models, response surfaces, Kriging, Gaussian processes, equivalent circuits, finite-element models, reduced-order models, machine learning, neural networks, physics-informed models, digital twins, multi-fidelity models, and multi-source fusion. Synonymous keywords within each category were combined via the Boolean operator “OR”, whereas transformer-related terms were paired with diagnostic or modeling terms using the Boolean operator “AND”. The standalone keyword “transformer” was avoided to prevent confusion with the attention-based Transformer architecture in artificial intelligence. Research adopting Transformer neural networks was included only if the physical research subject was a power transformer, and the corresponding model was designed and validated specifically for transformer fault diagnosis, condition evaluation or state prediction.

Screening of the retrieved literature was implemented sequentially through duplicate removal, title–abstract screening, full-text evaluation, and verification of citation context. Publications were included if they focused on power transformers or their closely related components, investigated fault diagnosis, condition assessment, internal state estimation, physical field reconstruction, lifetime prediction, uncertainty analysis or fault mechanism simulation, and provided clear descriptions of model inputs, outputs, data sources, modeling frameworks or validation schemes. Papers concerning irrelevant electrical equipment, general power system operation, wireless power transfer, renewable energy forecasting, or universal fault detection without transformer-specific datasets and experimental verification were excluded. General studies on surrogate modeling, reduced-order modeling, uncertainty quantification and explainable artificial intelligence were reserved merely as methodological references, rather than direct evidence supporting transformer diagnostic performance. The collected literature was categorized according to electrical and insulation faults, thermal faults, as well as mechanical and structural faults. A comparative analysis was further carried out among conventional approximation models, equivalent-circuit models, finite-element models, reduced-order models, data-driven models, physics-informed models, digital twins and hybrid surrogate models. Noticeably, the reviewed literature varies greatly in transformer parameters, fault definitions, data sources, experimental conditions and evaluation metrics. Accordingly, results are summarized via thematic classification and critical qualitative comparison.

## 3. Fault Characteristics and Diagnostic Information for Power Transformers

Power transformer faults usually result from the long-term interaction of electrical stress, thermal aging, mechanical deformation, and insulation degradation. Their external manifestations are reflected in different diagnostic information, such as dissolved-gas characteristics, partial-discharge signals, temperature responses, frequency-response changes, vibration or acoustic features, and routine electrical-test results [22]. These signals and tests have formed the basis of conventional transformer fault diagnosis and remain important for fault screening, condition evaluation, and maintenance decision-making. However, as fault evolution is often accompanied by multiphysical coupling, weak early-stage features, measurement noise, and operating-condition variations, a single diagnostic indicator or fixed empirical criterion is often insufficient to describe complex fault behavior [23]. Therefore, electrical and insulation-related faults, thermal faults, and mechanical or structural faults are analyzed according to their formation mechanisms, signal responses, data sources, and diagnostic challenges, providing a clearer fault-scenario basis for subsequent modeling analysis.

### 3.1. Electrical and Insulation-Related Fault Characteristics

Electrical and insulation-related faults include inter-turn and inter-layer short circuits, local insulation breakdown, partial discharge, abnormal grounding, dielectric aging, moisture accumulation, and conductive deterioration in oil–paper systems. These faults are driven by electric-field concentration, thermal aging, contamination, moisture migration, mechanical damage to insulation, and repeated transient overvoltage [24,25,26]. They may initially remain localized, but continued discharge or thermal decomposition can weaken dielectric strength, generate gases, carbonize insulation, and develop into winding or ground faults. Unlike mechanical deformation, which primarily changes structure and distributed parameters, the defining feature of this category is the deterioration of electrical conduction or dielectric integrity.

**Figure 1:**
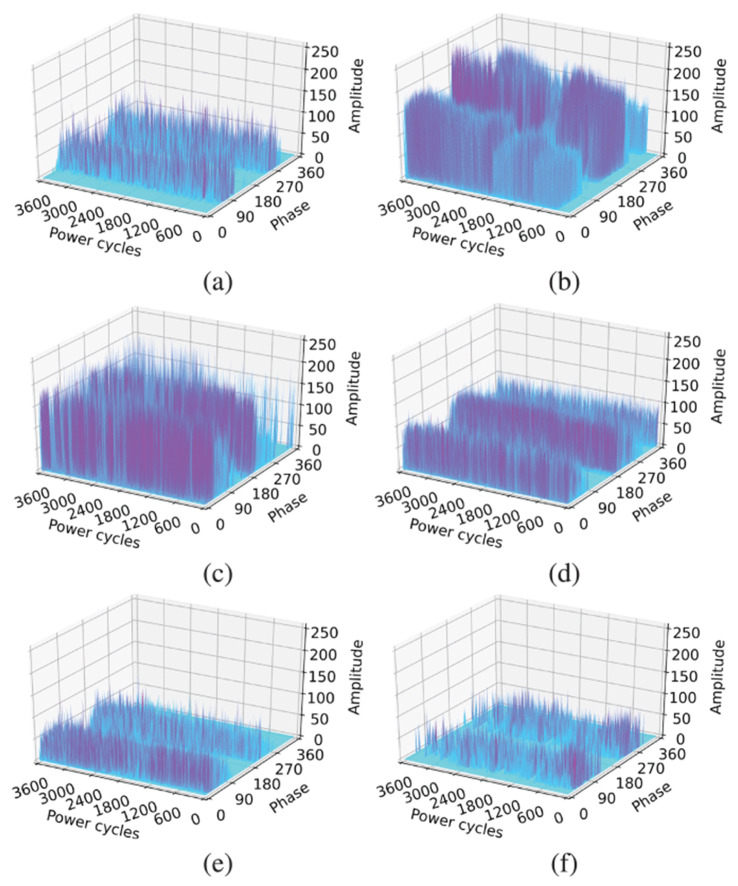
Representative sequential PRPD patterns for six typical transformer insulation-defect types: (**a**) Protruding electrode; (**b**) floating potential; (**c**) particle; (**d**) surface; (**e**) turn-to-turn; and (**f**) void discharge [27].

The corresponding diagnostic information is obtained from DGA, PD pulse patterns, dielectric loss, insulation resistance, frequency-domain spectroscopy (FDS), polarization and depolarization current (PDC), recovery-voltage measurements (RVM), winding resistance, excitation current, capacitance, and other electrical-response measurements. DGA provides chemically integrated evidence of oil and paper decomposition, whereas PD measurements provide faster information about active localized discharge. Figure 1 presents the sequential phase-resolved partial-discharge (PRPD) patterns of six typical insulation-defect types. The dataset covers the principal discharge-fault categories commonly encountered in oil–paper-insulated equipment and therefore provides a representative basis for cross-comparing the discharge characteristics of different defect modes. Differences in discharge phase, amplitude, and occurrence density provide intuitive evidence for identifying the corresponding discharge mechanisms [27]. Dielectric-response techniques are sensitive to moisture, aging, and polarization processes but are strongly affected by temperature and material heterogeneity. Zhou et al. [28] used a multiphysics model to characterize moisture transport in oil-impregnated paper, illustrating the need to relate measured dielectric behavior to internal moisture distribution. Studies using PD characteristics, local feature extraction, and electrical or vibration responses have also demonstrated the value of multi-domain information for longitudinal insulation faults [29,30,31].

The diagnostic difficulty is not a lack of candidate indicators but their non-unique and condition-dependent relationship with fault state. Gas concentrations depend on fault energy, oil volume, sampling interval, gas transport, and previous operating history. PD signals are affected by sensor bandwidth, propagation path, electromagnetic interference, and defect geometry. Dielectric parameters vary with temperature, moisture, aging degree, and test frequency. In addition, confirmed severe faults are rare, and maintenance labels may describe the final observed defect rather than the complete preceding degradation process. Electrical and insulation diagnosis therefore requires models that integrate complementary indicators, account for uncertainty and operating conditions, and avoid interpreting a single gas ratio, discharge pattern, or dielectric parameter as unique evidence of causality.

### 3.2. Thermal Fault Characteristics

Transformer thermal faults are mainly caused by abnormal losses in windings or cores, overload operation, poor electrical contact, insufficient heat dissipation, cooling-system degradation, oil-flow blockage, or structural defects. The resulting local temperature rise can accelerate oil–paper insulation aging, reduce dielectric strength, and further promote hotspot accumulation or insulation breakdown [32]. Unlike discharge-related faults that may exhibit transient or pulse-like responses, thermal faults generally evolve gradually and are closely influenced by load history, ambient temperature, cooling mode, oil circulation, winding losses, and core-loss distribution. Their observable responses therefore reflect the combined effects of heat generation, heat transfer, and operating-condition variation, while the most critical internal hotspots are often difficult to measure directly.

**Figure 2:**
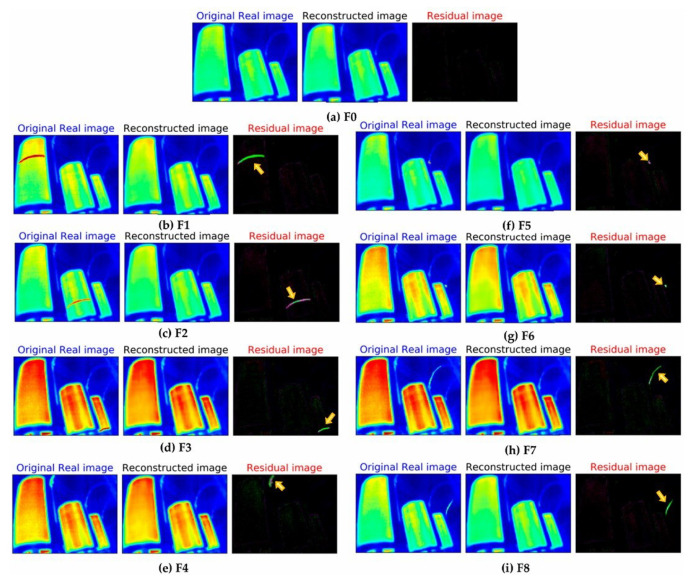
Real, reconstructed, and differential infrared thermal images under different transformer conditions. The arrows indicate the fault locations. In the real and reconstructed images, warmer and cooler colors indicate relatively higher and lower temperatures, respectively; in the residual images, brighter colors indicate larger pixel-wise differences, whereas dark regions indicate little or no difference [33].

Traditional thermal diagnosis mainly relies on operating records, oil-related indicators, contact temperature measurements, infrared thermography, and other non-contact sensing techniques. Load current, top-oil temperature, shell temperature, cooling-system status, and dissolved gases provide complementary information about thermal loading and overheating-related degradation. Infrared thermal imaging further enables rapid and non-invasive observation of external temperature distributions. As shown in Figure 2, Fanchiang et al. [33] used infrared thermal images to distinguish different transformer thermal conditions through the analysis of real, reconstructed, and differential images, demonstrating the value of spatial temperature information for identifying external overheating features. He et al. [34] developed a non-contact hotspot-temperature measurement method based on ultrasonic excitation, showing that internal thermal states may also be inferred through indirect response signals when direct sensor installation is impractical. These studies broaden the observability of transformer thermal faults beyond conventional point-temperature measurements. Nevertheless, infrared images mainly represent surface temperature distributions, whereas indirect hotspot measurements depend on signal propagation, system calibration, sensor arrangement, and the relationship between the measured response and the internal heat source.

**Figure 3:**
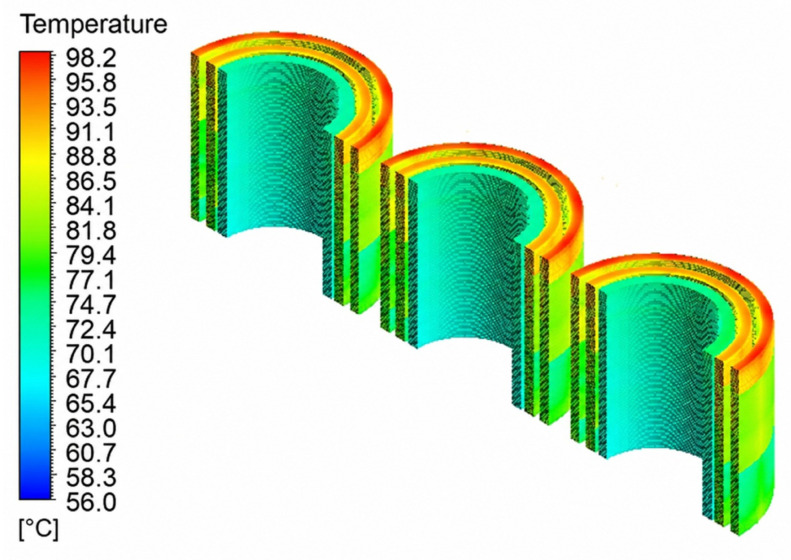
Temperature distribution of three-phase transformer windings obtained from electromagnetic–thermal–fluid coupling analysis [15].

Thermal Equivalent-Circuit (TEC) models and numerical simulations provide complementary physical tools for interpreting these measurements. TEC models represent heat-transfer processes through lumped thermal resistances and capacitances and are widely used for top-oil and winding-temperature estimation because of their clear physical meaning and relatively low computational cost. Zhang et al. [35] improved the IEC thermal model for oil-natural air-natural transformers by optimizing thermal parameters through dynamic simulation, indicating that parameter correction can enhance temperature estimation under changing operating conditions. Finite-element and computational-fluid-dynamics analyses provide more detailed descriptions of oil flow, heat dissipation, and spatial temperature distribution. Figure 3 presents a representative model of the internal temperature distribution in three-phase transformer windings obtained through electromagnetic–thermal–fluid coupling analysis. The spatial temperature gradients and localized hotspot regions revealed by the model complement external infrared observations and provide intuitive information for hotspot identification and cooling-system performance evaluation [15,36]. These physical approaches establish an important connection among transformer structure, operating conditions, and thermal responses. However, lumped models may not adequately represent localized or transient thermal behavior, and their parameters can vary with aging, oil condition, cooling performance, and ambient environment. High-fidelity simulations provide greater spatial detail but require accurate material properties, heat-transfer coefficients, geometric models, and boundary conditions, while their computational cost and validation requirements limit repeated or real-time application.

Overall, conventional temperature monitoring, oil–gas analysis, infrared thermography, equivalent thermal models, and numerical simulation provide a mature foundation for transformer thermal assessment. Monitoring techniques offer direct engineering evidence of thermal abnormalities, while physics-based models support the interpretation of heat-generation and heat-transfer processes. With increasingly variable loading and cooling conditions, however, thermal diagnosis is extending from the detection of apparent temperature abnormalities toward the estimation of hidden hotspots, transient temperature evolution, and cumulative insulation degradation. The main difficulty is therefore no longer limited to acquiring thermal information, but lies in consistently relating heterogeneous measurements, operating variables, and internal temperature states across different conditions. Suitable surrogate models may help address this requirement by preserving essential thermal relationships while reducing the computational and parameter-identification burden of detailed physical analysis.

### 3.3. Mechanical and Structural Fault Characteristics

Mechanical and structural faults include axial or radial winding deformation, winding displacement, clamping looseness, core or fastening displacement, and structural degradation induced by short-circuit forces, transport impacts, sustained vibration, and repeated thermal expansion [37]. Such faults modify the internal geometry, distributed inductance and capacitance, mechanical stiffness, resonance characteristics, and force-transmission paths of the transformer. These structural changes may further disturb the electromagnetic and thermal fields and, in severe cases, accelerate insulation deterioration. Accordingly, diagnostic analysis primarily focuses on evaluating structural integrity and mechanical stability, while the associated electrical and thermal responses serve as complementary indicators of fault development and severity.

**Figure 4:**
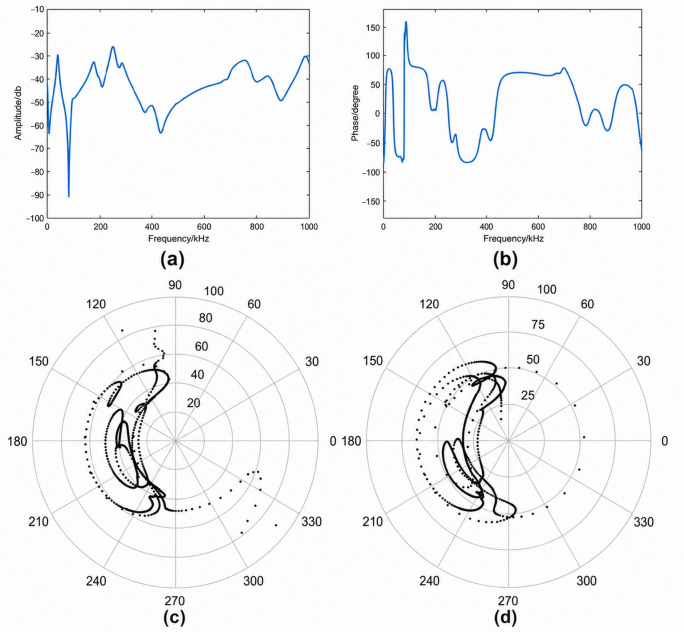
The frequency response of the healthy winding and typical FRA polar plots of windings: (**a**) amplitude-frequency signature of winding in normal condition; (**b**) phase-frequency signature of winding in healthy condition; (**c**) polar plot of winding in normal condition; (**d**) polar plot of winding in fault condition [39].

Frequency response analysis (FRA), short-circuit impedance testing, capacitance or inductance measurement, and equivalent-circuit interpretation are widely used for winding deformation. Structural changes modify distributed electrical parameters and consequently shift resonances or alter frequency-response magnitudes. Liu et al. [38] used finite-element-assisted FRA analysis to examine the influence of axial displacement and radial deformation. Liu et al. [39] further transformed FRA data into polar plots and extracted texture characteristics for distinguishing winding conditions, while Maulik et al. [40] related local mechanical damage to changes in winding parallel capacitance and driving-point impedance. Figure 4 presents representative winding frequency responses and their corresponding polar-coordinate representations. The polar transformation converts resonance-related magnitude variations into characteristic trajectory patterns, thereby facilitating the visual comparison of winding conditions and supporting texture-feature extraction for mechanical fault classification [39]. These approaches are non-invasive and physically meaningful, but their conclusions depend on connection leads, grounding, test configuration, manufacturing tolerance, and the choice of reference fingerprint. Similar curve variations may be produced by different deformation modes, so response matching alone does not guarantee unique localization or severity estimation.

Vibration and acoustic measurements provide complementary information about the dynamic structural behavior of transformers. The measured signals are jointly influenced by core magnetostriction, electromagnetic forces, winding motion, cooling devices, tank resonance, and structural transmission paths [41]. Yao et al. [42] analyzed core-related vibration and acoustic characteristics through multiphysics coupling, while Pei et al. [43] extracted wavelet-packet energy and Mel-frequency cepstral features to characterize acoustic responses. In parallel, FEM-based structural and electromagnetic analyses, including investigations of core grounding and coupled loss–temperature behavior [44], provide physical insights for interpreting vibration and acoustic signals. Nevertheless, sensor location, load level, propagation path, environmental noise, and overlapping excitation sources may obscure weak defect signatures. Reliable diagnosis therefore requires either carefully normalized physical indicators or models capable of separating asset-, operating-, and fault-related variations.

The three fault categories establish different diagnostic targets and information time scales. Electrical and insulation-related diagnosis emphasizes discharge, dielectric, gas, and electrical-integrity indicators; thermal diagnosis emphasizes load history, cooling state, temperature evolution, and hidden hotspots; and mechanical and structural diagnosis emphasizes distributed-parameter variation, vibration, acoustics, and geometry-related responses. The categories may interact physically, but their dominant diagnostic information is sufficiently distinct to avoid repeating the same methods in the model taxonomy. The following section therefore changes the organizing principle: it no longer revisits faults one by one, but compares how different surrogate-model families represent the mappings defined above.

## 4. Types and Development of Transformer Surrogate Models

Transformer fault diagnosis requires a clear correspondence between fault characteristics and modeling representations. Electrical, thermal, mechanical, and chemical faults arise from different physical processes and exhibit distinct response signals, feature patterns, and diagnostic targets, which determine the information basis for fault identification. Surrogate models provide simplified computational representations for these diagnostic relationships by approximating the mapping among measurable signals, operating conditions, physical responses, and fault states. With the increasing complexity of diagnostic tasks, transformer surrogate modeling has evolved from empirical approximation and explicit feature mapping to data-driven nonlinear representation, and further toward hybrid modeling that incorporates physical constraints or mechanism-related information.

Transformer surrogate models differ not only in their mathematical formulations, but also in the type of diagnostic information they represent, the physical knowledge they retain, and the computational resources they require. To avoid evaluating individual studies solely through reported diagnostic accuracy, the following sections compare traditional approximation, data-driven, and hybrid models in terms of representation capability, data dependence, physical interpretability, generalization, computational cost, and online applicability. These dimensions provide a consistent basis for relating model characteristics to transformer fault types, monitoring data, and engineering diagnostic requirements.

### 4.1. Traditional Approximation Models

Approximation models are used in transformer fault diagnosis to simplify complex electromagnetic, thermal, and mechanical processes and to relate fault-induced variations to measurable diagnostic responses. Based on physical laws and engineering equivalents, these models commonly include equivalent circuits, finite-element models, reduced-order models, empirical fitting, and lumped-parameter approximations [18,45,46]. Their key advantage lies in physical interpretability, which supports fault mechanism analysis, parameter identification, state estimation, and preliminary diagnosis [47]. Although they are less suited to high-dimensional data processing, online diagnosis, and adaptive intelligent assessment, their principles remain important for understanding fault evolution and developing physics-guided surrogate models.

#### 4.1.1. Equivalent-Circuit Model

Equivalent-Circuit Models represent the distributed electromagnetic behavior of transformers through lumped resistance, inductance, capacitance, and mutual-coupling parameters. They are particularly suitable for offline FRA interpretation, winding-parameter identification, and reference-based condition comparison, with winding deformation, winding displacement, and inter-turn short circuits being the most representative fault targets. Because these parameters are related to winding geometry, insulation condition, magnetic coupling, and losses, ECMs provide a physically interpretable mapping from internal changes to impedance, resonance, and transient electrical responses [48]. Mondal et al. [49], for example, developed a multistage ladder network to represent abnormal winding states. This type of model is particularly useful for FRA interpretation, winding-parameter estimation, and fault-sensitivity analysis because structural variations can be converted into circuit quantities that are directly comparable with measured responses.

The diagnostic capability of an ECM is determined by more than its circuit order. Increasing model order may improve frequency-domain fitting, but it also introduces correlated parameters and reduces identification stability. Moreover, different internal defects may produce similar changes in equivalent capacitance or inductance, so accurate response reproduction does not necessarily ensure unique fault localization. Parameter identification based on offline tests further restricts continuous application [50], although Dirik et al. [51] showed that winding parameters can also be estimated from operating measurements. ECMs are therefore most suitable when the dominant fault effect can be represented by a limited number of physically meaningful parameters. For localized insulation deterioration, fast transients, and strongly coupled multiphysics processes, they are better used as interpretable model components or physical priors than as complete diagnostic representations [52].

#### 4.1.2. Finite-Element Method

Within traditional approximation models, the Finite-Element Model (FEM) is adopted when detailed spatial field information is required for fault mechanism analysis. Its principal application scenarios include offline mechanism investigation, controlled fault simulation, and diagnostic-sensitivity analysis, especially for winding deformation, core grounding abnormalities, localized overheating, and cooling-related faults. By discretizing transformer geometry and solving electromagnetic, thermal, or structural field equations, FEM can describe the influence of faults on field distribution and system response with relatively high physical fidelity [53,54]. Therefore, it is particularly suitable for mechanism verification, sensitivity analysis, and fault simulation under complex structural, material, and operating conditions.

**Figure 5:**
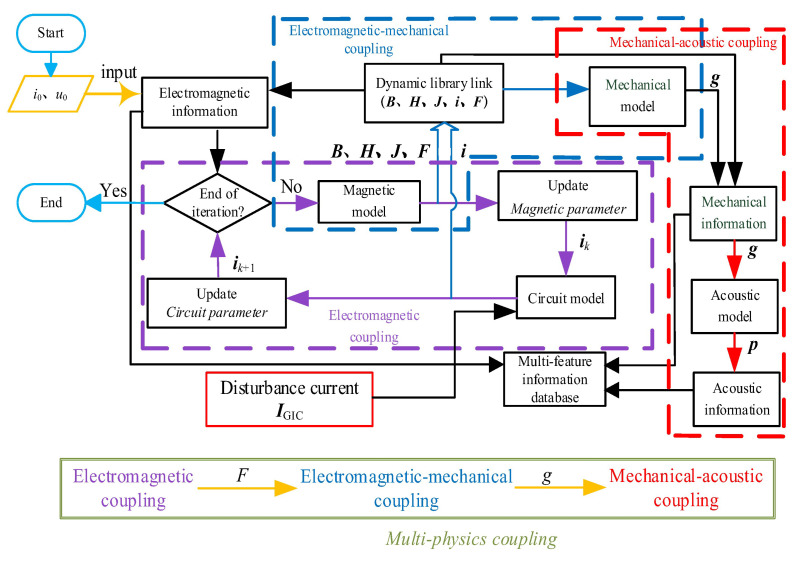
Multi-physics coupling model of the transformer. Purple, blue, and red denote electromagnetic coupling, electromagnetic–mechanical coupling, and mechanical–acoustic coupling, respectively. The yellow arrows indicate the transfer of electromagnetic force (F) and mechanical acceleration (g) between successive coupling stages [57].

In transformer fault diagnosis, FEM has been widely used to establish physical relationships between fault-induced structural or operating variations and measurable diagnostic features. Liu et al. [38] combined finite-element simulation with field tests to analyze how axial displacement and radial deformation of windings affect electrical parameters, and related simulation platforms have also been used to evaluate the diagnostic sensitivity of FRA and short-circuit impedance (SCI) testing [55,56]. Beyond winding deformation, FEM-based multiphysics models support the analysis of electromagnetic excitation, mechanical vibration, acoustic propagation, localized overheating, oil flow, and grounding-related fault processes. As shown in Figure 5, An et al. [57] established an electromagnetic–mechanical–acoustic coupling framework in which electromagnetic quantities are first calculated using finite-element analysis, the resulting electromagnetic forces are transferred to the mechanical model to determine component vibration, and the vibration responses are subsequently introduced into the acoustic model. This sequential coupling links winding current, magnetic flux density, electromagnetic force, vibration acceleration, and sound pressure within a unified physical framework, providing physically consistent multi-domain information for transformer condition analysis. Sun et al. [44] further applied electromagnetic–fluid–solid coupling modeling to investigate temperature distributions, oil-flow characteristics, and core grounding faults. These studies demonstrate that FEM provides high-fidelity fault-response simulations and establishes interpretable connections between internal physical processes and externally measurable diagnostic signals. Nevertheless, its dependence on detailed geometry, material parameters, boundary conditions, and computational resources limits direct online application, motivating its integration with reduced-order and data-driven surrogate models.

FEM should nevertheless be distinguished from a computationally efficient surrogate model. Its principal role in the surrogate-modeling framework is often to provide high-fidelity reference solutions, generate controlled fault samples, support parameter-sensitivity analysis, and calibrate simplified models. Detailed geometry, mesh resolution, nonlinear material properties, and boundary conditions substantially affect simulation accuracy, while practical parameters such as contact conditions, oil-flow coefficients, and aging-dependent material properties are often uncertain. The associated computational cost also limits repeated parameter inversion and online calculation. Consequently, FEM-assisted diagnosis should report both agreement with measurements and sensitivity to uncertain model inputs. Its integration with response surfaces, reduced-order methods, or data-driven mappings is generally more suitable for repeated diagnosis than direct execution of the full-order model [58,59].

#### 4.1.3. Reduced-Order Model

As high-fidelity physical models often involve high computational cost, the Reduced-Order Model (ROM) is developed to improve calculation efficiency while retaining the dominant characteristics of the original system. ROMs are therefore well suited to digital-twin updating, repeated parameter inversion, and near-real-time state reconstruction, particularly for transformer hotspots, cooling degradation, insulation thermal aging, and parameterizable winding faults. ROM projects high-dimensional physical models onto a low-dimensional representation or constructs simplified response mappings, thereby reducing computational burden while preserving key dynamic or field information [60]. Common ROM methods include Proper Orthogonal Decomposition (POD), modal truncation, frequency-domain reduction, Kriging response surfaces, and polynomial response surface approximations [60,61,62], which are suitable for rapid parameter scanning, inverse problem solving, and real-time diagnosis.

**Figure 6:**
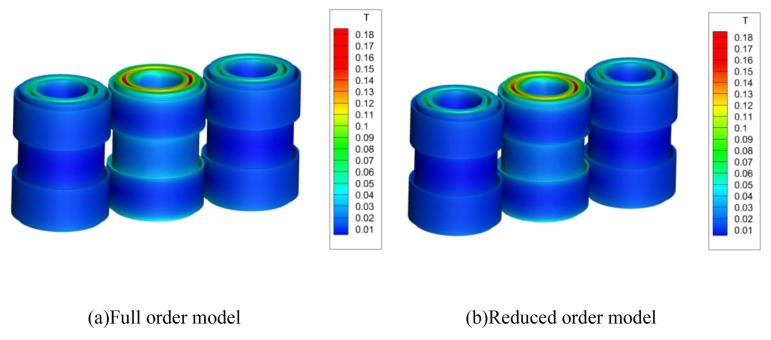
Magnetic field reduction model results [62].

In transformer applications, ROMs are mainly used to transform complex physical or circuit responses into computationally efficient representations for fault localization, thermal assessment, and field reconstruction. Shah et al. [63] synthesized driving-point impedance information into a low-order equivalent model, providing a simplified representation for winding fault localization. Zhang et al. [64] combined field measurements with three-dimensional numerical models in a reduced-order digital-twin framework for transformer hotspot temperature monitoring and prediction. Xu et al. [62] further integrated POD with Kriging response surfaces to reconstruct the winding magnetic field efficiently and accurately, as shown in Figure 6. These results demonstrate that ROMs can preserve greater physical consistency than black-box mappings while achieving substantially lower online cost than FEM. Their reliability, however, is normally strongest within the parameter and operating domains used to construct the reduced basis; previously unrepresented fault modes or strong nonlinear transitions may require basis enrichment or model reconstruction.

Taken together, ECM, FEM, and ROM form a hierarchy of physical representation. ECM provides the clearest parameter-level interpretation and the lowest computational cost, FEM offers the greatest spatial and multiphysics detail, and ROM provides a compromise between physical fidelity and repeated computational efficiency. Their common limitation is not simply insufficient nonlinear capability, but dependence on predefined structures, parameters, and operating domains. This restricts their ability to represent unknown fault patterns and heterogeneous monitoring information, motivating data-driven models that learn diagnostic mappings directly from observed or simulated data [65].

### 4.2. Data-Driven Diagnostic Models

Traditional approximation models provide structured and physically interpretable descriptions of transformer fault mechanisms, but their simplified assumptions and predefined parameter spaces are often insufficient for high-dimensional monitoring data, nonlinear feature interactions, and variable operating conditions. Data-driven models (DDMs) address these limitations by learning fault-related mappings directly from operational or simulation data, thereby reducing dependence on detailed physical formulations [66,67]. Their effectiveness, however, depends on the quality and representativeness of the available data. Transformer datasets commonly exhibit limited fault samples, class imbalance, uncertain labels, measurement noise, and incomplete coverage of operating conditions. Lopes et al. [68] examined the effects of imbalance and noise on DGA-based diagnosis, while Rao et al. [69] demonstrated the value of large multi-utility datasets in improving sample diversity. These factors jointly determine the reliability of the learned fault representations and provide the basis for selecting appropriate data-driven modeling strategies.

Within this framework, data-driven surrogate models differ mainly in how they represent diagnostic information and construct nonlinear mappings. Machine learning models are primarily applied to structured variables and extracted signal features, offering efficient model construction and relatively direct feature-level analysis. Neural network models extend this capability to images, sequences, spectra, and multimodal monitoring data, enabling more complex representations of transformer fault states. As the learned representations become increasingly complex, the relationship between model inputs and diagnostic outputs also becomes less explicit. The Interpretability of Data-Driven Diagnostic Models therefore complements these modeling approaches by revealing influential variables, signal regions, and decision evidence, thereby improving the transparency and credibility of data-driven surrogate diagnosis.

#### 4.2.1. Machine Learning Models

**Figure 7:**
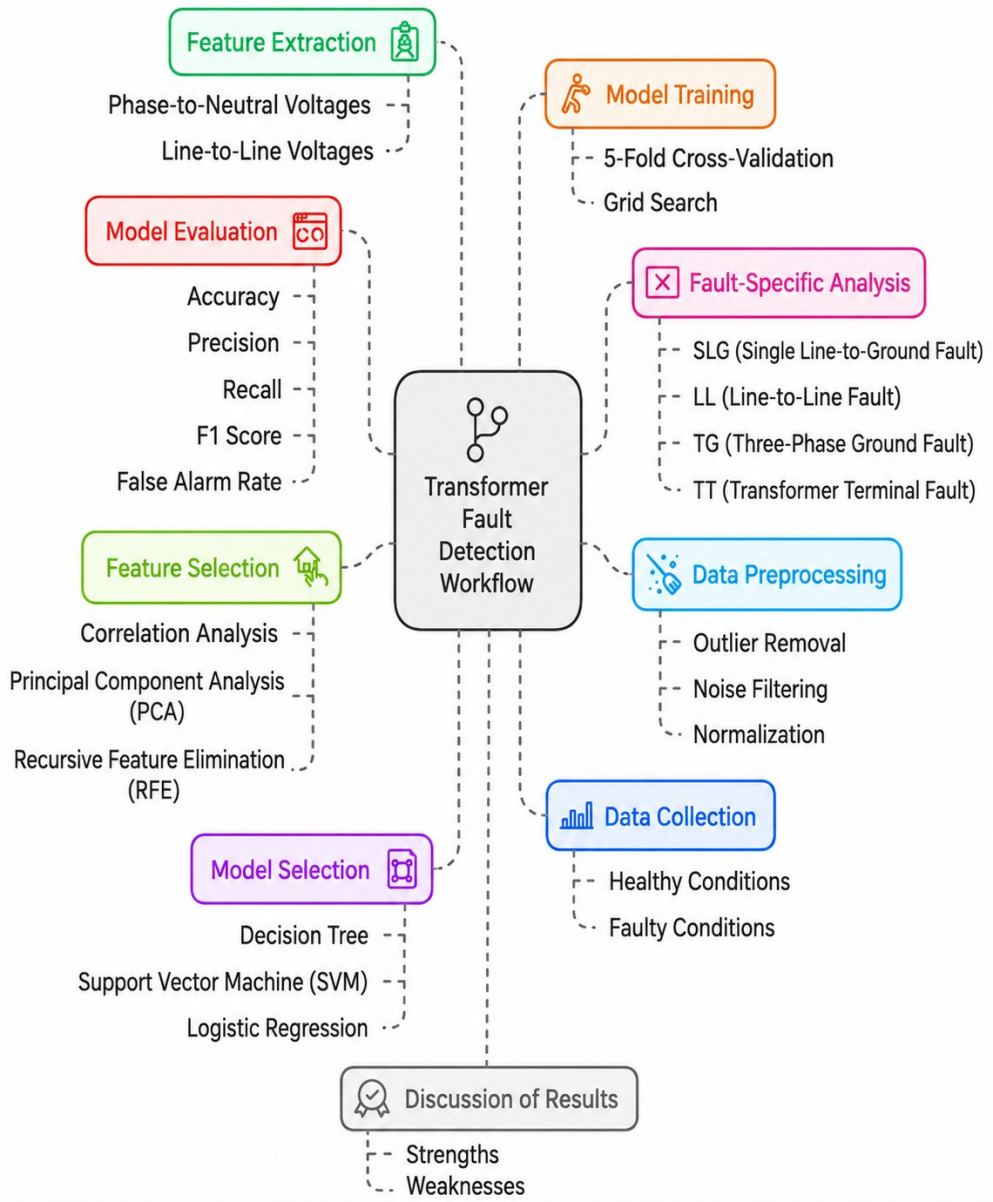
Representative machine learning workflow for transformer fault detection [72].

Machine learning models have become important data-driven surrogate modeling tools for transformer fault diagnosis, as they can learn nonlinear relationships between monitoring features and fault states without explicitly constructing detailed physical models. They are particularly appropriate for routine screening based on structured DGA, SCADA, oil-condition, FRA, vibration, and electrical-response features, with discharge, overheating, insulation deterioration, winding deformation, and terminal faults as the main diagnostic targets. Typical methods, such as Support Vector Machines (SVMs), Decision Trees (DTs), Random Forests (RFs), and Logistic Regression (LR), can extract discriminative information from dissolved-gas, frequency-response, vibration, voltage, and operational data, making them suitable for fault identification, severity assessment, and state prediction [70]. For DGA-based diagnosis, Zhang et al. [71] combined information-entropy-based feature weighting with a fuzzy SVM to enhance gas-feature separability and improve the classification of similar fault types. Figure 7 presents a representative machine learning workflow for transformer fault detection based on phase-to-neutral and line-to-line voltage measurements. The framework integrates data collection under healthy and faulty conditions, signal preprocessing, feature extraction and selection, classifier selection, model training, cross-validation, and fault-specific performance evaluation. This structured workflow illustrates how raw electrical measurements are progressively transformed into fault-discriminative representations and provides a common basis for comparing different classifiers across multiple transformer fault conditions [72]. These studies indicate that machine learning models can transform complex monitoring signals into fault-discriminative representations, thereby improving diagnostic efficiency and classification performance under nonlinear and multi-feature conditions.

Beyond basic fault classification, machine learning-based surrogate models have also been extended to diagnostic robustness, parameter optimization, and state prediction. By integrating feature engineering, hyperparameter tuning, and optimization algorithms, these models can improve their adaptability to noisy monitoring data, nonlinear feature relationships, and limited samples. For example, Soliman et al. [73] constructed classification models using operational and oil-condition parameters, such as dissolved gases, acid value, and moisture content, showing that optimized feature–model configurations can enhance diagnostic stability and generalization. Related studies have further applied optimization-assisted machine learning to structural design and temperature trend prediction, indicating that data-driven surrogate models can support not only fault identification but also performance evaluation and operational decision-making. These results demonstrate the flexibility of machine learning models in extracting useful diagnostic information from heterogeneous transformer data.

Nevertheless, the effectiveness of machine learning-based surrogate models is closely related to sample independence, label confidence, class balance, signal quality, and coverage of operating conditions rather than dataset size alone. Noise robustness established by adding artificial perturbations does not fully represent field uncertainty caused by sensor drift, missing intervals, maintenance interventions, mixed faults, and changing loads. Similarly, random record-level division can overestimate generalization when measurements from the same transformer or fault episode occur in both training and test sets. Studies should therefore report the numbers of transformers and independent fault events in addition to sample count, document the label source and preprocessing decisions, describe class distributions and operating ranges, and use transformer-wise or time-separated validation whenever possible [68,69]. Accuracy should be accompanied by class-wise recall, specificity, precision, F1 score, confusion matrices, and uncertainty or calibration measures, particularly when rare fault classes have greater engineering consequences. When operating conditions or transformer populations differ from the training data, adaptation or transfer-learning strategies may be required, but their reliability must still be verified with an independent target-domain test set [70].

#### 4.2.2. Neural Network Models

Neural network models are an important class of data-driven surrogate models because they can establish nonlinear mappings between transformer monitoring features and fault states under complex operating conditions [74]. Their main application scenarios involve automated analysis of raw or weakly processed PRPD patterns, vibration sequences, acoustic signals, electrical waveforms, and time–frequency images, making them especially suitable for partial discharge, inter-turn short circuits, winding looseness, and core-related mechanical abnormalities. Compared with conventional machine learning methods, neural networks have stronger representation ability and are suitable for extracting fault-related information from current, voltage, vibration, frequency-response, and discharge signals. Early studies mainly adopted shallow architectures, such as Back Propagation (BP) neural networks and Multi-Layer Perceptron (MLP), to identify winding deformation, inter-turn short circuits, and other typical faults. For example, Jin et al. [75] improved BP-based fault diagnosis by combining residual modules, feature fusion, and SVM-based discrimination, while Liu et al. [48] used frequency-response indicators and an MLP model to locate winding inter-disk distance variation faults. These studies indicate that shallow neural networks can enhance nonlinear fault representation, but their performance still depends on manually designed features and the quality of diagnostic indicators [76].

**Figure 8:**
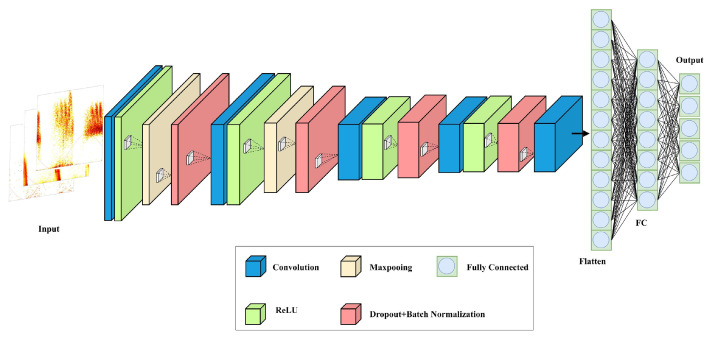
Proposed CNN architecture. The white cubes represent the local receptive fields, or processing windows [27].

With the growth of computing power and transformer monitoring data, deep neural networks have been increasingly applied to feature extraction and fault pattern recognition. Convolutional Neural Networks (CNNs) and Recurrent Neural Networks (RNNs) can automatically learn hierarchical spatial or temporal features from raw monitoring signals, reducing the dependence on handcrafted feature construction [77]. Do et al. [27] applied a CNN to classify six types of discharge defects based on Phase-Resolved Partial Discharge (PRPD) signals, demonstrating its ability to learn discriminative discharge patterns, as shown in Figure 8. Zollanvari et al. [78] further used a deep RNN to capture temporal dependencies in vibration signals for early fault prediction. Overall, neural network-based surrogate models improve the representation of nonlinear and time-varying fault features, but their diagnostic reliability still depends on data scale, sample quality, and model generalization under changing operating conditions.

Neural networks reduce reliance on handcrafted features and can represent complex fault signatures, but greater model capacity does not guarantee stronger engineering performance. When datasets are small, highly correlated, or collected under limited operating conditions, deep models may learn transformer-specific or measurement-specific patterns rather than transferable fault characteristics. Their advantage over conventional machine learning should therefore be demonstrated using common datasets, independent asset-level validation, and ablation of feature-learning components. Model size, training variability, class imbalance, and inference cost should also be reported. Neural networks are most suitable when sufficient independent data are available and the diagnostic information is embedded in raw or weakly processed signals; otherwise, simpler feature-based models may provide comparable performance with lower computational and interpretive cost [17].

#### 4.2.3. Interpretability of Data-Driven Surrogate Models

Data-driven transformer diagnostic models can achieve high diagnostic accuracy, but their decision processes are often difficult to trace, which limits the credibility and engineering applicability of their outputs. Interpretability is particularly important for maintenance-oriented decision support and the verification of ambiguous DGA, FRA, PRPD, or vibration-based predictions, especially when electrical, thermal, and insulation-related fault indicators overlap. Explainability methods are therefore introduced to reveal how diagnostic variables, signal features, and local regions contribute to model predictions. Global explanations characterize the overall importance of different inputs, whereas local explanations clarify the evidence supporting individual diagnostic decisions. By comparing these results across data subsets, model configurations, and operating conditions, explainability analysis can further assess whether the model consistently captures fault-related characteristics rather than incidental correlations associated with transformer type, loading state, or measurement environment [79,80,81,82].

For data-driven surrogate models based on structured diagnostic variables, explainability should characterize not only which features are important but also how their values influence model outputs at the global and individual-sample levels. Feature importance, permutation analysis, sensitivity analysis, partial-dependence analysis, Local Interpretable Model-agnostic Explanations (LIME), and SHapley Additive exPlanations (SHAP) are commonly used for this purpose. Lundberg et al. [83] showed that SHAP-based analysis can aggregate local feature contributions to reveal the global structure and interaction patterns of tree-based models, while Barredo Arrieta et al. [79] systematically summarized the applicability of model-specific and model-agnostic explanation methods. In transformer diagnosis, these techniques can quantify the contributions of dissolved gases, gas ratios, oil-condition indices, thermal variables, and extracted electrical features, thereby identifying the principal evidence supporting a diagnostic result. They improve the transparency of feature-based surrogate models and facilitate the comparison of diagnostic indicators. However, strong correlations among gases, derived ratios, and operating variables may redistribute attribution values, and feature contribution should not be interpreted directly as fault causality [82].

Deep learning surrogate models require further interpretation of the spatial, temporal, and spectral representations extracted from diagnostic signals. Montavon et al. [84] analyzed gradient-based attribution, relevance propagation, and perturbation methods for interpreting deep neural networks, whereas Samek et al. [85] reviewed saliency maps, class-activation mapping, integrated gradients, and related representation-level techniques. These methods are applicable to CNN- and RNN-based transformer diagnostic models, such as the PRPD classification model developed by Do et al. [27] and the vibration-based early-fault prediction model reported by Zollanvari et al. [78]. By locating influential phase–amplitude regions, frequency components, or temporal intervals, they help determine whether model predictions are supported by fault-relevant signal characteristics. Such visualization and attribution improve the traceability of deep diagnostic models, although the resulting maps may vary with network parameters, reference inputs, and perturbation settings and should therefore be examined using feature masking and repeated- model comparisons.

Explainability methods enable data-driven surrogate models to reveal the features, signal regions, and variable contributions underlying their diagnostic outputs. This improves the transparency and credibility of model predictions and provides a basis for identifying influential fault indicators and guiding diagnostic analysis. However, such methods remain largely post hoc and cannot guarantee that the learned relationships are physically consistent. This limitation motivates the further integration of physical information and mechanism-based constraints in hybrid surrogate models.

### 4.3. Hybrid Surrogate Models

Hybrid surrogate models are developed to overcome the limitations of single physical models and purely data-driven approaches by combining physical knowledge, monitoring data, and intelligent learning strategies. This integration helps improve diagnostic accuracy while maintaining physical consistency, computational efficiency, and robustness under complex operating conditions and multi-fault coupling scenarios [65,86,87]. According to the fusion mechanism, current hybrid surrogate models mainly follow two technical paths. One incorporates physical constraints, such as governing equations or mechanism-based priors, into model training to enhance interpretability and physical consistency. The other integrates multi-source or multi-fidelity information to exploit complementary features from simulations, monitoring data, and operational records, thereby improving diagnostic reliability and generalization.

#### 4.3.1. Physics-Informed Neural Networks

Physics-informed neural networks (PINNs) represent an important physics-guided surrogate modeling strategy for transformer fault diagnosis. They are most applicable to sensor-sparse state reconstruction and digital-twin prediction, particularly for winding hotspots, cooling abnormalities, thermal aging, and other faults governed by continuous thermal or electromagnetic processes. By incorporating governing equations, such as electromagnetic field equations, structural mechanics equations, or heat conduction equations, into the network loss function, PINNs constrain model learning with both data information and physical mechanisms. This enables the model to improve prediction accuracy while maintaining physical consistency, interpretability, and robustness, which is particularly valuable for transformer fault processes involving thermal diffusion, electromagnetic coupling, and structural degradation.

**Figure 9:**
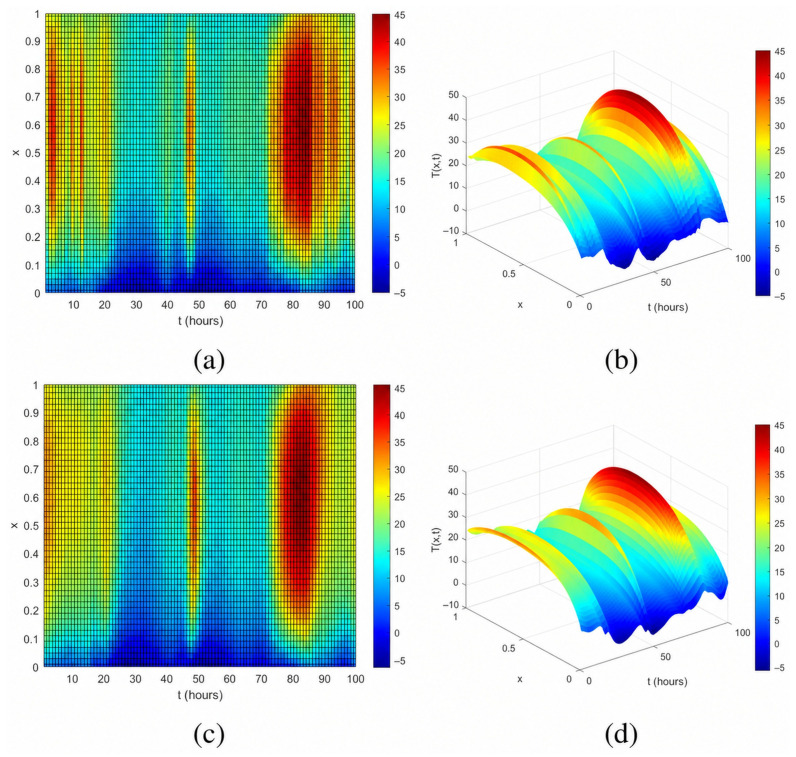
Temperature distributions obtained using the FVM and predicted by the PINN for the first 100 h of the dataset: (**a**) two-dimensional color map of the FVM solution; (**b**) three-dimensional surface plot of the FVM solution; (**c**) two-dimensional color map of the PINN prediction; and (**d**) three-dimensional surface plot of the PINN prediction [86].

In transformer applications, PINNs have mainly been explored for thermal field prediction, insulation aging assessment, and spatiotemporal state estimation. Bragone et al. [86] embedded the heat diffusion equation into neural network training to model transformer thermal characteristics. Figure 9 presents a representative time-series comparison between the reference solutions obtained using the Finite Volume Method (FVM) and the corresponding thermal responses predicted by the PINN during the first 100 h. The comparison directly illustrates the ability of the physics-informed model to reproduce dynamic transformer thermal behavior and provides a clear basis for evaluating temporal prediction consistency and deviations under changing operating conditions [86]. To address complex spatiotemporal coupling, Ramirez et al. [88] further combined physics-based partial differential equations with neural networks for winding-temperature and insulation-aging estimation and introduced a residual-based attention mechanism to improve convergence and computational efficiency. These studies demonstrate that PINNs can integrate physical modeling with data-driven learning, supporting computationally efficient and physically constrained transformer state prediction.

Nevertheless, the practical performance of PINN-based surrogate models still depends on data quality, boundary-condition accuracy, and the completeness of embedded physical constraints. Simulation or equation residuals can regularize a model under limited measurements, but they do not replace experimental or operational validation when geometry, material parameters, cooling conditions, or aging states are uncertain. Under small-sample, noisy, or cross-condition scenarios, additional strategies such as transfer learning, feature reconstruction, and data augmentation are often required to improve generalization. For example, Gramian Angular Summation Field-based transfer learning and Conditional Generative Adversarial Network-based sample generation have been used to enrich dissolved-gas feature representation and alleviate data imbalance problems [89,90]. The origin of generated and measured samples should remain traceable so that performance gains can be evaluated separately on simulated, laboratory, and field subsets.

#### 4.3.2. Multi-Fidelity and Multi-Source Data Fusion Models

Multi-fidelity and multi-source data fusion models extend transformer fault diagnosis beyond single-source learning by integrating complementary information from different sensing modalities, operating records, experiments, and simulations. They are particularly valuable for critical-transformer assessment and weak or coupled fault diagnosis, including thermal–insulation degradation, PD accompanied by dissolved-gas changes, winding deformation with vibration and FRA responses, and core faults associated with temperature and acoustic abnormalities. Although these two approaches are closely related, they address different aspects of information integration. Multi-source fusion combines heterogeneous measurements such as DGA, Supervisory Control and Data Acquisition (SCADA) variables, temperature, vibration, and maintenance records to provide a more comprehensive description of transformer condition. Multi-fidelity modeling instead combines data with different levels of accuracy, computational cost, and physical detail, including simplified analytical models, finite-element simulations, controlled experiments, and field measurements. By exploiting the complementarity among these data, both approaches can improve fault-state coverage, reduce dependence on any single information source, and enhance the robustness of surrogate models. Their effectiveness, however, depends on the appropriate treatment of source-specific bias, label uncertainty, sampling differences, and operating-domain mismatch, rather than on the simple accumulation of data volume.

**Figure 10:**
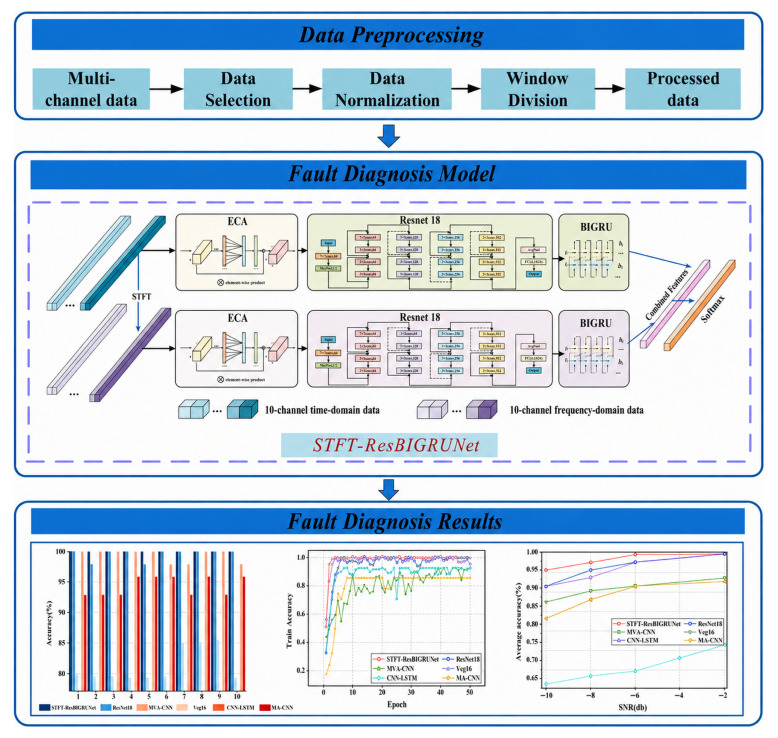
Overall multimodal fault-diagnosis framework based on multi-channel data preprocessing and the STFT-ResBiGRUNet model [92].

The effectiveness of multi-source fusion depends not on the number of incorporated variables alone, but on whether different information sources provide complementary descriptions of transformer condition and are properly aligned in time and feature space. Sensor-level fusion improves fault observability, while feature- and decision-level fusion strengthen the representation and integration of heterogeneous diagnostic evidence. Duz et al. [91] combined high-fidelity multi-sensor online monitoring with machine learning, showing that broader sensing coverage can improve fault-state discrimination. Yuan et al. [92] integrated electrical and environmental parameters through a Short-Time Fourier Transform (STFT)-ResBiGRUNet with attention, extending fusion to joint temporal–frequency representation learning, as illustrated in Figure 10. Wang et al. [93] further combined SCADA data, dissolved-gas measurements, and maintenance records, linking real-time operating information with slower condition indicators. These studies demonstrate that complementary sources can improve diagnostic completeness and robustness when a single modality cannot fully characterize transformer condition. However, the reported gains may arise from additional information, fusion architecture, or preprocessing, and these effects are seldom distinguished. Source-wise ablation and missing-channel evaluation are therefore necessary to quantify the contribution of each source and verify model reliability under incomplete measurements.

Although multi-source data fusion can improve diagnostic accuracy and robustness, its effectiveness depends on the consistency, alignment, and transferability of the integrated information. Simulation data can support mechanism coverage and surrogate pretraining, laboratory data can provide controlled calibration and fault discrimination, and operational data can be used for final external validation. Performance should also be evaluated for each source separately, because pooling all data before random partitioning may allow the model to exploit source-specific signatures rather than fault-related characteristics. Source-wise ablation, leave-one-transformer or leave-one-site-out validation, and testing under missing or degraded sensor channels are therefore important for determining whether fusion genuinely improves generalization. Moreover, accurate fusion does not necessarily ensure that the learned relationships are physically meaningful. Integrating multi-source information with physical constraints can improve both predictive performance and model interpretability [94]. Accordingly, transformer surrogate modeling is gradually evolving from isolated physical approximation or purely data-driven learning toward hybrid frameworks that combine physical knowledge, multi-source information, and intelligent algorithms, thereby providing stronger support for fault prediction, condition assessment, and maintenance decision-making [95].

### 4.4. Online Diagnosis and Deployment Requirements

The computational efficiency of surrogate models provides an important basis for online transformer diagnosis, but inference speed alone does not determine practical deployability. A complete diagnostic cycle includes data acquisition, synchronization, preprocessing, communication, model inference, confidence assessment, and alarm generation. The total processing time of this chain should remain within the required diagnostic update interval.

(1) $$T_{\mathrm{acq}}+T_{\mathrm{pre}}+T_{\mathrm{comm}}+T_{\inf}+T_{\mathrm{dec}}\leq T_{\mathrm{update}},$$

Here, $T_{\mathrm{acq}}$, $T_{\mathrm{pre}}$, $T_{\mathrm{comm}}$, $T_{\inf}$, and $T_{\mathrm{dec}}$ denote the acquisition, preprocessing, communication, inference, and decision times, respectively, while $T_{\mathrm{update}}$ represents the required diagnostic update interval. This relation distinguishes model-level inference efficiency from the end-to-end latency of the complete monitoring process. Online-oriented studies should therefore report preprocessing cost, communication delay, inference time, throughput, and total response time rather than describing a model as “real-time” solely on the basis of single-sample inference.

Measurement and model-update frequencies should be matched to the temporal characteristics of the monitored information. SCADA variables, load, temperature, and cooling states are generally suitable for periodic state estimation, whereas PD, vibration, acoustic, and other high-frequency signals require continuous acquisition followed by window-based filtering, feature extraction, or local representation learning. DGA and insulation-aging indicators evolve more slowly and are therefore more appropriate for trend assessment. Raw sampling frequency should consequently be distinguished from diagnostic update frequency, which is determined jointly by the signal-analysis window, feature-generation interval, communication cycle, and required alarm response. When heterogeneous measurements are integrated, asynchronous sampling, time-stamp alignment, missing intervals, delayed data, and changes in sensor configuration must also be considered [91,93]. These differences indicate that comprehensive diagnosis cannot be implemented simply by collecting all available signals at the same frequency.

Comprehensive transformer diagnosis is technically feasible, but the continuous installation of all available sensing modalities on every transformer is neither economically necessary nor operationally realistic. A practical monitoring system should adopt a hierarchical and risk-oriented sensing strategy. Routinely available SCADA, load, temperature, and basic condition information can support continuous state screening, while online DGA may be added for important units or where insulation-related risks require closer observation. PD, vibration, acoustic, and high-frequency electrical monitoring can then be selectively deployed for critical assets, specific fault concerns, or secondary diagnosis after abnormal trends are detected. Comprehensive diagnosis should therefore be understood as the coordinated use of complementary information at different monitoring levels rather than the simultaneous deployment of the maximum number of sensors. Such a configuration can balance diagnostic coverage and confidence against installation cost, communication load, sensor maintenance, and data-management complexity.

Based on this hierarchical sensing strategy, the deployment architecture should be selected according to asset criticality, data volume, diagnostic urgency, communication conditions, and model complexity. Edge devices can perform local preprocessing, event screening, and rapid inference for high-frequency signals, thereby reducing communication requirements and preserving basic diagnostic capability during network interruption. Station-level servers can integrate SCADA, DGA, thermal, and other condition information for coordinated assessment, while cloud or control-center platforms are more suitable for long-term storage, fleet-level comparison, and model updating. ECMs, ROMs, and conventional machine learning models are generally compatible with industrial CPUs or embedded processors. Neural networks may require pruning, quantization, knowledge distillation, or hardware acceleration, whereas PINNs and other hybrid models should be evaluated according to the inference cost of the trained surrogate rather than their offline training cost. Multi-source systems should additionally retain fallback operation when individual sensing channels or communication links become unavailable. Processor type, model size, memory consumption, inference latency, and dependence on specialized hardware should therefore be reported explicitly.

Finally, online validation should reproduce continuous field operation rather than rely only on randomly partitioned historical data. Time-ordered, leave-one-transformer-out, and leave-one-site-out evaluations are more representative of practical transfer because they reduce information leakage among correlated records. Detection delay, false-alarm and missed-alarm rates, confidence calibration, sensor-drift sensitivity, missing-channel robustness, and long-term availability should accompany conventional accuracy metrics. Low-confidence outputs should trigger repeated measurement, complementary testing, or expert review rather than deterministic alarm decisions. Overall, traditional approximation, data-driven, and hybrid surrogate models can all support online diagnosis when their sensing requirements, computational latency, system interfaces, and reliability are matched to the target application. The practical objective is therefore not to maximize model complexity or sensor quantity, but to construct a sufficiently informative, computationally efficient, and maintainable diagnostic chain.

### 4.5. Comparative Synthesis and Future Directions

The preceding analysis shows that transformer surrogate modeling has gradually evolved from simplified physical approximation toward data-driven, physics-informed, and multi-source-integrated frameworks. Traditional approximation models retain clear parameter correspondence and remain valuable for mechanism analysis, sensitivity evaluation, and controlled simulation. Data-driven models extend diagnostic capability to high-dimensional monitoring data and nonlinear fault characteristics, while hybrid models seek to combine computational efficiency with physical consistency. Together with the online deployment requirements discussed above, these developments indicate that the practical value of a surrogate model depends not only on predictive performance, but also on its data requirements, interpretability, transferability, computational cost, and compatibility with existing monitoring systems.

Table 1 provides a section-level synthesis of these model families by linking their representation basis and diagnostic use to their principal strengths, limitations, validation needs, and most appropriate deployment roles.

The maturity of current approaches nevertheless varies across diagnostic tasks. Models based on DGA, SCADA variables, oil-condition indices, and extracted electrical features generally have relatively clear input structures and are closer to operational implementation. By contrast, models using raw PRPD patterns, vibration sequences, multiphysics fields, or heterogeneous sensing information provide stronger representation capability but require more extensive data, computation, and validation. In our view, the principal contribution of advanced surrogate modeling is therefore not simply an improvement in classification accuracy. Its greater value lies in connecting different forms of diagnostic information, reducing repeated high-fidelity computation, and enabling rapid estimation of internal transformer states that cannot be measured directly.

Several limitations remain evident across the reviewed studies. Many models are developed using laboratory measurements, numerical simulations, or repeated records from a limited number of transformers, whereas independent validation across transformer designs, sites, and operating periods remains insufficient. Reported performance is also commonly dominated by accuracy-based metrics, with less attention to uncertainty, physical residuals, computational latency, missing-channel robustness, and long-term model drift. For physics-informed, multi-fidelity, and digital-twin-oriented approaches, improvements demonstrated under controlled conditions have not yet been consistently verified through continuous field operation. These limitations make it difficult to determine whether the learned relationships represent transferable fault characteristics or remain dependent on specific datasets and experimental settings.

Future research should therefore move from isolated model development toward task-oriented and deployment-oriented surrogate frameworks. Model selection should be guided by the diagnostic objective, available sensing information, required physical interpretation, fault-evolution time scale, and hardware environment rather than by algorithm complexity alone. Physically based models remain appropriate for parameter inversion and mechanism analysis, data-driven models are advantageous for complex monitoring patterns, and hybrid models offer greater potential when reliable physical knowledge and heterogeneous operational data are both available. Further progress requires standardized cross-transformer validation, uncertainty-aware prediction, adaptive updating under data drift, quantitative evaluation of physical consistency, and coordinated integration with SCADA, edge-computing, and maintenance decision systems. The preferred surrogate should ultimately be the least complex model that can satisfy the required diagnostic accuracy, physical credibility, robustness, and response time.

## 5. Conclusions

This paper systematically reviews the development and application of surrogate models for transformer fault diagnosis. Starting from the characteristics and observable responses of electrical and insulation-related, thermal, and mechanical faults, the reviewed studies were organized according to traditional approximation, data-driven, and hybrid modeling strategies. The analysis shows that these model families provide different but complementary capabilities. Traditional approximation models retain clear physical relationships and remain valuable for mechanism analysis and parameter estimation, whereas data-driven models offer greater flexibility in representing nonlinear relationships contained in monitoring data. Hybrid models further combine physical knowledge, simulation information, and heterogeneous measurements, providing a promising approach for improving diagnostic reliability under complex operating conditions. Overall, the suitability of a surrogate model depends on the fault characteristics, available diagnostic information, modeling objective, and engineering requirements rather than on predictive accuracy alone.

Despite the progress achieved, several issues still require further investigation. The limited availability of representative and independently validated fault data continues to restrict model generalization, particularly across transformer types, operating conditions, and monitoring environments. Future studies should therefore strengthen data quality control, cross-condition validation, and uncertainty analysis, while developing more consistent evaluation frameworks for comparing different surrogate approaches. Greater attention should also be given to the integration of physical mechanisms with data-driven learning, especially for fault states that are difficult to observe directly or are represented by sparse measurements. In addition, multi-source modeling should move beyond the simple accumulation of sensing variables toward effective information alignment, contribution assessment, and reliable operation under incomplete data. Finally, lightweight inference, adaptive model updating, and integration with existing monitoring and SCADA platforms will be important for translating surrogate models from offline studies into practical online diagnosis. These directions are expected to support the development of more reliable, interpretable, and engineering-oriented transformer diagnostic systems.

## Figures and Tables

**Table 1:** Comparison of surrogate-model families for power transformer fault diagnosis.

Model Family	Modeling Basis and Diagnostic Inputs	Strengths	Limitations	Application Scenarios
Equivalent-circuit model (ECM) [48,49,51,52]	Lumped resistance, inductance, capacitance, and mutual-coupling parameters derived from FRA or impedance responses.	Direct parameter correspondence, low computational cost, and interpretable sensitivity analysis.	Non-unique parameter–fault mapping; possible instability in high-order identification; limited localized and multiphysics fidelity.	Winding deformation, displacement, and inter-turn-fault analysis; offline FRA interpretation and parameter estimation.
Finite-element model (FEM) [38,44,55,56,57,58,59]	Discretized electromagnetic, thermal, fluid, and structural field equations using geometry, material properties, and boundary conditions.	High physical and spatial fidelity; supports mechanism verification and controlled data generation.	Strong dependence on geometry, materials, meshes, and boundary conditions; high computational cost; calibration is required.	Offline analysis of winding deformation, core grounding, hotspots, and cooling faults; controlled simulation and high-fidelity data generation.
Reduced-order model (ROM) [60,61,62,63,64]	Low-dimensional projections or response surfaces constructed from high-fidelity field, temperature, or impedance data.	Retains dominant physical behavior while enabling rapid repeated evaluation.	Validity is strongest inside the sampled domain; new fault modes or nonlinear transitions may require basis enrichment.	Near-real-time hotspot or field reconstruction, parameter inversion, and digital-twin updating within the sampled domain.
Conventional machine learning [70,71,72]	Feature-based mappings learned from DGA, SCADA, oil-condition, FRA, vibration, and operating variables.	Flexible nonlinear mapping with moderate computational demand; effective for structured features.	Sensitive to label noise, class imbalance, sensor drift, and record-level leakage; limited physical interpretation.	Routine fault classification, severity assessment, and trend screening using structured condition data on edge or station processors.
Deep neural network [27,77,78]	Hierarchical representations learned directly from raw or weakly processed PRPD, vibration, acoustic, waveform, and time–frequency data.	Automated hierarchical feature learning and strong spatial or temporal representation.	Requires sufficient independent data; risks overfitting and opaque decisions; inference cost can be substantial.	Automated recognition of partial-discharge, winding, and core-related faults when sufficiently large cross-asset datasets are available.
Physics-informed neural network (PINN) [86,88]	Neural networks constrained by governing-equation residuals, initial or boundary conditions, and sparse measured or simulated data.	Physical regularization and sensor-sparse reconstruction of continuous internal states.	Sensitive to equation, parameter, and boundary-condition misspecification; training and field validation remain challenging.	Sensor-sparse thermal or electromagnetic state estimation, physically constrained reconstruction, and digital-twin updating.
Multi-fidelity and multi-source fusion [91,92,93,94]	Joint representation of heterogeneous SCADA, DGA, temperature, vibration, maintenance, simulation, laboratory, and field data.	Broadens observability and exploits complementary sources when one channel is insufficient.	Requires temporal alignment, source-bias control, missing-channel testing, and source-wise ablation.	Critical-asset monitoring, coupled-fault assessment, and coordinated multimodal diagnosis under missing or asynchronous channels.

## Data Availability

All data generated or analysed during this study are included in this published article.
